# Evidence-Based Practice Implementation in a Pediatric Inpatient Unit: Nurses’ Experiences of Organizational Change

**DOI:** 10.3390/nursrep16060183

**Published:** 2026-05-27

**Authors:** Cânia Torres, Francisco Mendes, Paula Duarte, Maria Barbieri-Figueiredo

**Affiliations:** 1 School of Medicine and Biomedical Sciences, University of Porto (ICBAS), Rua de Jorge Viterbo Ferreira 228, 4050-313 Porto, Portugal; caniatorres@esep.up.pt; 2 RISE-Health, Nursing School, University of Porto (ESEP), Rua Dr. Bernardino de Almeida, 4200-072 Porto, Portugal; 3 Department of Pediatrics, São João Local Health Unit (ULS São João), Alameda Professor Hernâni Monteiro, 4200-319 Porto, Portugal; francisco.mendes@ulssjoao.min-saude.pt (F.M.); paula.alexandra.duarte@ulssjoao.min-saude.pt (P.D.)

**Keywords:** evidence-based practice, knowledge-to-action framework, pediatric nursing, sustainability, implementation science

## Abstract

**Background:** Sustained implementation of evidence-based practice (EBP) remains a challenge in complex clinical environments. Structured knowledge translation frameworks, such as the Knowledge-to-Action model (KTA), have been proposed to facilitate integration of evidence into care. However, less is known about how nurses experience the consolidation of KTA-guided implementation in clinical practice. This study aimed to explore nurses’ experiences of changes in clinical practice and professional development following consolidation of KTA guided implementation in a pediatric inpatient unit. **Methods:** A qualitative descriptive study was conducted using two focus groups with 20 pediatric nurses who had participated in the implementation process. One year after consolidation of the implementation process, data were collected via videoconference using a semi-structured interview guide and analyzed through thematic content analysis following Bardin’s methodological framework. **Results:** Four themes emerged: (1) contribution of the KTA to the clinical context; (2) influence on EBP competencies; (3) impact on professional development; and (4) sustainability within the organizational context. The implementation was experienced as a shift in care philosophy, with evidence becoming central to clinical reasoning, team alignment, and service structures. **Conclusions:** Participants perceived that the implementation of the KTA framework extended beyond competence development, influencing clinical practice, professional interactions, and organizational routines within the unit. Sustainability was associated with contextual adaptation, leadership alignment, and structural integration of evidence-based practices into care. These findings contribute to understanding mechanisms supporting durable EBP implementation in pediatric settings.

## 1. Introduction

Healthcare systems are increasingly characterized by clinical complexity, rapid knowledge production, and growing expectations regarding quality, safety, and accountability. Within this landscape, evidence-based practice (EBP) has become a central pillar of contemporary nursing, supporting clinical decision-making that integrates research evidence, professional expertise, and patient and family preferences [1,2]. Despite its recognized relevance, the integration of evidence into nursing practice remains inconsistent across clinical settings.

Research over the past decade has shown that challenges associated with EBP adoption extend beyond individual knowledge or skills. Institutional factors including leadership engagement, workload pressures, structural constraints, and workplace culture play a decisive role in shaping the uptake and sustainability of evidence-informed practices [3,4]. Although educational strategies and professional development initiatives have been widely implemented, these approaches alone have proven insufficient to secure sustained change in clinical practice [5]. Increasingly, researchers argue that structured implementation processes are required to embed evidence within everyday care and service systems rather than relying solely on individual motivation or competence.

In response to this need, knowledge translation (KT) frameworks have been developed to guide the systematic integration of research into practice. The Knowledge-to-Action (KTA) framework combines knowledge creation with an iterative action cycle that supports contextual adaptation, implementation of tailored strategies, monitoring, and sustained knowledge use [6]. By structuring the process from problem identification to long-term integration, the action cycle provides a mechanism for translating individual learning into practice-level change. In this unit, the KTA framework was operationalized through a structured implementation process that included locally driven problem identification, adaptation of evidence, and development of monitoring mechanisms [7].

The cyclical and context-sensitive nature of the KTA framework makes it particularly relevant for understanding how implementation processes evolve beyond initial adoption and become integrated into routine clinical practice. However, despite the increasing use of knowledge translation frameworks such as the KTA model, existing literature has predominantly focused on early implementation phases and short-term outcomes [6,8]. Consequently, limited evidence exists regarding how nurses experience the consolidation and ongoing incorporation of evidence-informed practices within complex healthcare settings over time [9,10,11]. This gap is especially relevant in pediatric inpatient units, where care intensity, clinical variability, and the central role of families may influence the durability of practice changes [12].

Previous publications from this broader implementation project addressed the implementation protocol [7] and barriers and facilitators influencing evidence-based practice implementation in pediatric nursing care during earlier phases of the process [13]. In contrast, the present study focuses specifically on nurses’ experiences following consolidation of the implementation, understood as the phase following completion of the structured implementation process, during which evidence-based activities and organizational mechanisms remained active within routine clinical practice. In this study, sustainability refers to participants’ perceptions regarding the continued incorporation of evidence-based activities and organizational structures into everyday clinical practice over time.

Accordingly, this study aimed to explore nurses’ experiences of changes in clinical practice and professional development after the consolidation of KTA implementation in a pediatric inpatient unit, with a particular focus on perceptions of integration and ongoing incorporation of evidence-based practices into clinical and organizational contexts.

## 2. Materials and Methods

In this study, consolidation refers to the phase following completion of the structured implementation process in which evidence-based activities and organizational mechanisms remained active within routine clinical practice.

### 2.1. Study Design

This study adopted a qualitative descriptive design and was conducted and reported in accordance with the Consolidated Criteria for Reporting Qualitative Research (COREQ) checklist [14].

### 2.2. Setting and Participants

The study was conducted in a pediatric inpatient unit of a tertiary university hospital in northern Portugal that provides acute and complex care to children and their families. The unit provides multidisciplinary care and acts as a regional referral center. During the implementation period, the unit underwent an internal reorganization that included the merging of two previously separate nursing teams.

Purposive sampling was used to recruit nurses who had actively participated in all stages of the KTA implementation. Twenty nurses, corresponding to 62.5% of those involved in the implementation activities (N = 32), participated in two focus groups conducted on 6 and 13 March 2023.

Eligibility criteria included direct involvement in the implementation activities and at least one year of professional experience within the unit during the consolidation phase. This ensured participants had sufficient exposure to reflect meaningfully on changes in clinical practice and professional development.

The KTA implementation had been conducted over a multi-year period (2019–2022) prior to data collection and involved structured and participatory strategies aimed at integrating evidence into clinical practice.

Clinical priorities addressed during implementation included standardization of family-centered care practices, revision of nursing documentation and clinical indicators, strengthening of evidence-informed decision-making processes, and promotion of collaborative reflection regarding pediatric care practices. Structured mechanisms introduced during this period included regular journal clubs, working groups focused on care improvement, revision of care procedures based on current evidence, and internally led research activities incorporated into everyday clinical practice.

The initiative was supported by formal nursing leadership, facilitating alignment between evidence-based activities and service priorities. By the time of data collection, implementation activities and organizational structures associated with the KTA process remained active within the unit’s clinical and organizational context.

### 2.3. Data Collection

A semi-structured interview guide was developed to explore nurses’ perceptions of organizational changes following KTA implementation. The guide was informed by literature on evidence-based practice implementation and addressed four domains: perceived organizational changes, influence on professional roles and practice, leadership involvement, and sustainability of implemented structures [15,16,17]. Examples of guiding questions included:

“What contribution did the implementation of the KTA framework have in your professional context?”

“How did the implementation influence your clinical practice?”

“How do you perceive the sustainability of the model?”

The guide was organized into three phases: introduction, development, and closure. The introductory phase included presentation of participants and researchers, clarification of study objectives, explanation of moderator and observer roles, confirmation of confidentiality, authorization for audio recording, and completion of the sociodemographic form and informed consent. Participants were also informed that divergent opinions and critical perspectives were equally valuable for the study in order to reduce the influence of social desirability during data collection.

The development phase consisted of open-ended questions designed to facilitate in-depth discussion of participants’ experiences. The closing phase included a summary of key discussion points, allowing participants to clarify or confirm interpretations (member checking at session level).

Focus groups were conducted via Zoom videoconferencing software (Zoom Video Communications, San Jose, CA, USA; Available online: https://zoom.us/; accessed on 10 January 2025) at times convenient for participants. Each session lasted approximately 90 min, was audio-recorded with consent, and transcribed verbatim. Focus groups were conducted in Portuguese and quotations were translated into English by the research team with attention to preservation of meaning.

The sessions were moderated by the principal researcher, who has clinical experience in pediatric nursing and prior experience conducting focus groups, and supervised by a senior PhD researcher with expertise in qualitative methodology. The principal researcher had previous professional contact with some participants through the implementation context but did not occupy a direct managerial role within the unit during data collection. Given the researchers’ involvement in the broader implementation process, reflexive discussions were conducted throughout analysis to critically consider how prior assumptions, professional experiences, and expectations could influence interpretation of the findings. The focus group format enabled exploration of shared perspectives, identification of patterns, and examination of divergent views within the group dynamic.

### 2.4. Data Analysis

All sessions were audio-recorded with prior consent and transcribed verbatim. Transcripts were reviewed for accuracy before analysis. Qualitative data were organized using NVivo software version 12 (QSR International, Melbourne, Australia) to facilitate systematic coding, data management, and traceability of analytical decisions.

Thematic content analysis was conducted following Bardin’s three-phase methodological framework [18]: (1) pre-analysis; (2) exploration of the material; and (3) treatment of results with inference and interpretation. During the pre-analysis phase, repeated readings of the transcripts were undertaken to support immersion in the data and identification of preliminary analytical directions. In the exploration phase, meaning units were identified and coded systematically across transcripts. Codes were subsequently grouped into categories and subcategories based on shared semantic and conceptual characteristics. In the final phase, categories were interpreted in relation to the study objectives and implementation context.

Two researchers independently reviewed and coded the transcripts before discussing categories collaboratively within the research team. Divergences in interpretation were resolved through discussion and consensus. Analytical memos and reflexive discussions were used throughout the process to support transparency and critical examination of emerging interpretations.

Participants were anonymized using a code consisting of the letter “E” followed by a sequential number (e.g., E1, E2). Sociodemographic characteristics were analyzed descriptively to contextualize the findings.

Data sufficiency was considered achieved through thematic repetition and coherence across focus groups, with no substantially new categories emerging in the second group discussion. Considerations regarding sample adequacy were also informed by the concept of information power, given the specificity of participants’ experiences and the focused aim of the study.

To enhance credibility, summaries of key discussion points were presented to participants at the end of each focus group session to allow clarification and confirmation of interpretations.

### 2.5. Rigor

Rigor was addressed through strategies embedded in the study design and analytical process. Participants were selected based on direct involvement in the KTA implementation process, ensuring experiential relevance and depth of insight. The inclusion of most eligible nurses within the unit strengthened the contextual robustness of the findings.

During data collection, summaries of key discussion points were presented to participants at the end of each focus group to allow clarification and confirmation of interpretations. This supported alignment between participants’ intended meanings and the researchers’ understanding.

Throughout analysis, coding and category development were discussed within the research team to support consistency and critical examination of interpretations. The structured application of Bardin’s analytical framework [18] provided a systematic pathway from raw data to thematic organization.

Detailed description of the setting, participants, and implementation context was provided to enable readers to assess the relevance of findings to other clinical environments.

### 2.6. Ethical Statement

The study adhered to established ethical principles. Ethical approval was obtained from the Ethics Committee for Health and from the Research Unit of the institution where the study was conducted (approval no. 93/2021).

Participants received written information about the study objectives and procedures and were given opportunities to ask questions prior to participation. Written informed consent was obtained before data collection. Participation was voluntary, and participants were informed of their right to withdraw at any time without consequence.

Focus group sessions were conducted through a password-protected virtual platform to ensure confidentiality. The study was conducted in accordance with applicable ethical and regulatory standards to safeguard participants’ rights and well-being.

## 3. Findings

Twenty nurses participated in the study. All participants were female, reflecting the gender composition of the nursing staff in the unit, and all held a specialization in pediatric nursing. Most had between 11 and 20 years of professional experience, although five participants had 21 or more years of experience. All reported prior training in evidence-based practice, research, and project implementation (Table 1).

### 3.1. Nurses’ Perceptions of Practice Transformation Following KTA Implementation

Analysis resulted in four main categories reflecting nurses’ perceptions of practice-level changes following implementation of the KTA model: (1) contribution of the KTA model to the clinical context; (2) influence on EBP competencies; (3) impact on professional development; and (4) sustainability of the model in the organizational context. Participants’ accounts suggested a progressive process in which engagement with evidence gradually influenced clinical reasoning, professional interactions, and service organization. Table 2 presents the categories and subcategories identified during analysis.

#### 3.1.1. Contribution of the KTA Model to the Clinical Context

Participants described changes at multiple levels of the clinical environment, including family care, individual professional practice, and service organization. Engagement with evidence was perceived as gradually reshaping the way clinical decisions were discussed and justified within the team:

*“There was a complete paradigm shift, where our practices started to be influenced by evidence (…) the care we provide to children and families gained more quality.”* (E14)

The implementation process was also described as facilitating team integration during a period of organizational transition, when two previously separate teams were merged:

*“This process brought the team together, at a time when two teams were merging, it was crucial.”* (E11)

At the service level, structural reorganization was emphasized:

*“The service reorganized itself in many aspects, which was very helpful for everyone.”* (E3)

and

*“The unit is very different now, more organized, and nurses are more concerned about these aspects.”* (E7)

#### 3.1.2. Influence of the KTA Model on EBP Competencies

This theme reflects changes in clinical reasoning, research knowledge, and professional interactions associated with the development of EBP competencies.

Participants described more explicit and systematic critical reflection:

*“We reflect critically on what we do (…) and whether it is the best for the child and family.”* (E6)

and

*“Before, we acted automatically; now, we stop to reflect critically on what we do and whether it is truly the best for each child and family.”* (E9)

Critical reflection and greater consideration of evidence during decision-making were recurrently described.

For some participants, involvement in research represented a significant shift in professional identity and engagement with evidence-informed practice:

*“I didn’t even know what those three words meant (EBP), now I’m even a researcher (…) incredible.”* (E2)

Research engagement was also associated with increased confidence and participation in evidence-informed clinical discussions.

Evidence also appeared to influence team communication and reduce variability in clinical decision-making. Participants described greater alignment in discussions regarding care practices:

*“There are discussions we no longer have because evidence wins.”* (E1)

and

*“now we discuss based on evidence…”* (E10)

Evidence was increasingly described as a shared reference supporting greater consistency in clinical reasoning and collaborative decision-making within the team.

Participants associated these changes with greater consistency in care practices and improved communication within the team:

*“Communication improved among everyone because care is more uniform.”* (E2)

and

*“Communication was undoubtedly facilitated because the standardization of procedures helped make this process easier.”* (E13)

For many participants, evidence-based practice evolved into an integrated way of working:

*“It became a new way of working (…) it’s really a philosophy we adopted.”* (E5)

Participants described greater consistency in discussions and decision-making within the unit.

#### 3.1.3. Impact on Professional Development

In contrast to competence-related changes, this theme reflects the subjective and relational dimensions of the implementation experience, particularly regarding professional identity, satisfaction, and sense of belonging within the team.

Participants described increased professional fulfilment and pride associated with involvement in evidence-based activities:

*“I feel proud of what this team achieved (…) I feel happier and more fulfilled at work.”* (E18)

Nursing leadership emerged as an important source of motivation and organizational support during implementation:

*“Our head nurse was very important (…) motivating us daily.”* (E2)

and

*“The nurse manager was undoubtedly a major facilitator of this process. She helped us understand that this dimension of nursing practice is also important.”* (E16)

The implementation experience was frequently perceived as personally and professionally transformative:

*“This project changed us at a personal and professional level.”* (E1)

The implementation process was associated with changes in professional satisfaction, motivation, and perceptions of participants’ roles within the team.

### 3.2. Sustainability of the Model in the Organizational Context

Participants expressed confidence in the continuity of the structures introduced during implementation, particularly journal clubs and research activities. However, they also recognized that maintaining these initiatives requires continued engagement from the team and ongoing organizational support. Journal clubs, research projects, and working groups were seen as mechanisms that help keep practice up to date:

*“The journal club, research projects and working groups are here to continue (…) it’s not possible to provide good care any other way.”* (E7)

Participants perceived journal clubs, research activities, and collaborative working groups as becoming integrated into the organizational routines of the unit, supporting continuity of evidence-based activities over time.

Continuity of structures was also emphasized:

*“I have no doubt it will remain forever and spread to other services.”* (E19)

Although participants generally described the implementation process positively, some acknowledged challenges associated with maintaining evidence-based activities alongside the demanding workload typical of pediatric inpatient care:

*“(…) children have very complex conditions, and sometimes it is not easy to balance that with this project, but having participation in journal clubs and scientific activities recognized as working time is very motivating.”* (E4)

and

*“(…) because of the complexity of care provided in this unit, it is sometimes difficult to balance clinical demands with this project.”* (E15)

Sustaining participation in journal clubs, research activities, and collaborative initiatives was described as requiring continued support and engagement from both the organization and the team.

## 4. Discussion

Engagement with evidence-based practice appeared to extend beyond the adoption of isolated interventions and was perceived as influencing clinical reasoning, professional interactions, and organizational dynamics within the pediatric inpatient unit. Evidence-informed activities progressively became incorporated into everyday clinical work, collaborative practices, and team communication following consolidation of the KTA implementation.

This study contributes to current implementation literature by providing empirical insight into how nurses experience the consolidation of evidence-based practice beyond early implementation phases or short-term outcomes. In this context, sustainability was perceived not simply as continuation of isolated initiatives, but as the ongoing incorporation of evidence-informed practices into professional and organizational life.

Participants described a process in which locally identified clinical problems, contextual adaptation of evidence, and structured mechanisms such as journal clubs and research initiatives supported the incorporation of evidence into everyday practice. Rather than functioning as isolated implementation activities, these elements were perceived as contributing to the normalization of evidence-informed reasoning within clinical decision-making and professional interactions. While these findings align with key components of the KTA cycle, particularly its emphasis on contextual adaptation and iterative implementation [6,8], they also illustrate how these processes may become integrated into everyday clinical and organizational contexts over time [11].

Sustainability was described not as the continuation of a project, but as the progressive integration of evidence-informed decision-making into everyday professional practice. Rather than resulting from simple protocol adoption, it emerged through continued engagement with the action cycle. This perception aligns with current understandings of sustainability as integration within practice and complex care environments [11,19]. This transition appears to have occurred through the gradual integration of evidence into team discussions and clinical decision-making processes, rather than through the immediate adoption of protocols. Evidence was portrayed as a reference point guiding clinical reasoning and reducing variability in decision-making. As one nurse stated, “there are discussions we no longer have because evidence wins,” suggesting that evidence increasingly served as a shared reference point within team interactions. Similar patterns have been described in the literature, where evidence-based practice contributes to greater consistency and alignment across professionals [20,21].

Strengthened critical-reflective thinking and increased involvement in research activities emerged as important dimensions of the implementation experience. The transition from limited familiarity with EBP to active engagement in research reflects previous studies showing that structured implementation initiatives can enhance research literacy and professional confidence [16,22]. The fact that the process was grounded in locally identified clinical problems may have contributed to this engagement, as problem-focused approaches are associated with greater relevance and ownership [19]. Leadership was consistently described as a facilitating factor. The role of the nurse manager was considered important for maintaining motivation and supporting alignment between evidence-based activities and daily work. Previous research has identified leadership as a determinant of EBP adoption and maintenance [21,23], particularly when leaders integrate evidence use into expectations and professional development processes. In this context, sustainability was not perceived as the continuation of a project but rather as the integration of specific mechanisms into practice.

The incorporation of structured mechanisms, such as journal clubs and research groups, into practice was described as supporting the maintenance of updated care. These structures were perceived as contributing to the maintenance of updated and differentiated care. Contemporary implementation research similarly frames maintenance of evidence-based activities as the integration of practices into everyday systems and professional norms rather than the preservation of time-limited projects [11]. Despite high clinical demands typical of pediatric inpatient settings, participants perceived evidence-based activities as becoming integrated into routine practice. This suggests that alignment between team engagement, leadership support, and structured mechanisms may contribute to the durability of evidence-informed practice.

Beyond these changes in practice, participants described increased pride and professional fulfilment. Engagement with research and evidence use appeared to strengthen professional identity. Previous studies have also associated research engagement with enhanced professional legitimacy and development [22,24]. When EBP aligns with both patient care and professional growth, sustained commitment may be reinforced.

An additional contextual element influencing participants’ experiences was the organizational restructuring occurring within the unit during the same period. The merging of two previously separate nursing teams may have contributed to greater alignment, communication, and collective identity independently of the implementation process itself. In this context, KTA-related activities may also have functioned as mechanisms supporting team integration during organizational transition, rather than solely influencing evidence-based practice implementation [15,21].

Although participants generally described the implementation process positively, sustaining evidence-based activities over time was also perceived as requiring continued organizational support and active engagement from the team. The demanding clinical workload characteristic of pediatric inpatient care was acknowledged as a contextual challenge for maintaining participation in journal clubs, research activities, and collaborative initiatives. In this context, participants described organizational measures intended to facilitate continued engagement with evidence-based activities. Journal clubs, training activities, organization of scientific events, and participation in scientific meetings were institutionally recognized as part of clinical working time, which may have supported the ongoing integration of these activities into professional practice.

These findings reinforce the understanding that sustainability within healthcare settings is dynamic, context-dependent, and reliant on continued organizational investment rather than automatically guaranteed following implementation [9,10,11].

At the same time, the findings should be interpreted with caution. All participants had been directly involved in the implementation process and had previous training in evidence-based practice, research, and project implementation, which may have contributed to particularly engaged and favorable perspectives toward the process. Less engaged or more skeptical professionals may have perceived the experience differently. In addition, the group-based nature of focus groups may have limited the expression of more divergent or critical views. Therefore, the findings reflect the experiences of a highly involved group within a specific organizational context and should not be interpreted as objective evidence of long-term organizational transformation or fully demonstrated integration.

Rather than demonstrating sustainability as a fixed organizational outcome, the findings suggest that normalization of evidence-informed practice may be experienced as the gradual normalization of evidence-informed reasoning within professional identity, team communication, and organizational expectations. This perspective contributes to current implementation literature by highlighting the relational and cultural dimensions through which evidence-based practices may become integrated into routine clinical work over time [10,11].

## 5. Conclusions

The findings suggest that the implementation of the KTA framework extended beyond individual competency development, influencing clinical reasoning, professional interactions, and care processes within the pediatric inpatient unit. Participants described evidence-based practice as becoming progressively integrated into everyday team routines and organizational dynamics.

Sustainability was perceived not as the continuation of a time-limited project, but as the ongoing incorporation of evidence-informed decision-making into professional practice, supported by leadership engagement and organizational structures such as journal clubs and research activities.

The study contributes an empirical perspective on how nurses experience the consolidation of evidence-based practice following KTA implementation in a complex pediatric clinical setting. The findings further suggest that integration may involve the gradual normalization of evidence-informed reasoning within professional relationships, team communication, and everyday clinical work.

However, these findings reflect the perceptions of a highly engaged group within a specific organizational context and should be interpreted with caution. This study did not include longitudinal evaluation or objective organizational indicators capable of demonstrating long-term normalization of evidence-informed practice or measurable organizational transformation.

### 5.1. Implications and Future Research

The findings suggest that sustained implementation of evidence-based practice may be facilitated by organizational strategies that integrate evidence-informed activities into everyday clinical work. Structured mechanisms such as journal clubs, collaborative reflection, and locally driven research initiatives were perceived as supporting continued engagement with evidence within the pediatric inpatient setting.

Leadership support and institutional recognition of evidence-based activities as part of clinical working time also emerged as important organizational conditions supporting ongoing implementation efforts.

Future research should explore how evidence-based activities become maintained and normalized within clinical teams over time and across different healthcare contexts. Longitudinal and mixed-methods studies may contribute to understanding how organizational conditions, professional engagement, and clinical demands influence the durability of evidence-informed practice following implementation processes.

### 5.2. Limitations

This study has several limitations. First, it was conducted within a single pediatric inpatient unit, which may limit transferability to other clinical contexts with different organizational cultures, leadership structures, or team dynamics. However, detailed contextual description was provided to support readers in assessing applicability to similar settings.

Participants had been directly involved in the implementation process, all possessed previous training in evidence-based practice, research, and project implementation, and all were female pediatric nurses, reflecting the composition of the unit. This context may have contributed to predominantly positive perceptions of the experience and limited the emergence of more critical or divergent perspectives. Although efforts were made to encourage open discussion during focus groups, the possibility of social desirability bias and conformity effects within group discussions cannot be excluded. Nurses who were less engaged in the implementation process or less available to participate may have experienced the process differently. These characteristics may also limit transferability to other professional and organizational contexts.

Finally, data were collected one year after consolidation of the implementation process. Although this allowed exploration of experiences beyond initial implementation, the study did not include longitudinal evaluation, clinical outcome indicators, or organizational audits capable of objectively assessing long-term sustainability. Future longitudinal and mixed-methods research could strengthen understanding of the measurable and sustained impact of evidence-based practice implementation over time.

## Figures and Tables

**Table 1:** Sociodemographic and professional characteristics of participants.

Characteristic	n (%)
Age (years)	
23–30	2 (10%)
31–35	8 (40%)
36–40	5 (25%)
41–50	5 (25%)
Gender	
Female	20 (100%)
Male	0 (0%)
Academic Qualification	
Bachelor’s degree	13 (65%)
Master’s degree	7 (35%)
Doctorate	0 (0%)
Pediatric Nursing Specialisation	
Yes	20 (100%)
No	0 (0%)
Years of Professional Experience	
3–10	1 (5%)
11–15	7 (35%)
16–20	7 (35%)
≥21	5 (25%)
Continuing Education	
Evidence-Based Practice	20 (100%)
Research	20 (100%)
Project Methodology/Implementation	20 (100%)
Management	2 (10%)

**Table 2:** Categories and subcategories emerging from focus group analysis.

Categories	Subcategories
Contribution of the KTA to the clinical context	Improved family involvement in care
	Strengthening of nurses’ clinical practice
	Contributions to service organization
Influence of the KTA on EBP competencies	Promotion of critical-reflective thinking
	Development of research knowledge
	Improved relationship management
	Improved peer communication
	Adoption of new work methodologies
Impact on professional development	Greater job satisfaction
	Leadership support
	Positive perception of the process
	Personal gains
Sustainability of the model in the organizational context	Structural contributions
	Organizational contributions

## Data Availability

The data presented in this study are not publicly available due to ethical and confidentiality constraints.

## Public Involvement Statement

There was no public or patient involvement in the design, conduct, reporting, or dissemination plans of this research.

## Guidelines and Standards Statement

This manuscript was prepared and reported in accordance with the Consolidated Criteria for Reporting Qualitative Research (COREQ) checklist [14].

## Abbreviations

The following abbreviations are used in this manuscript:

EBP	Evidence-Based Practice
KT	Knowledge Translation
KTA	Knowledge-to-Action
COREQ	Consolidated Criteria for Reporting Qualitative Research

## References

[1] Connor L., Dean J., McNett M., Tydings D.M., Shrout A., Gorsuch P.F., Hole A., Moore L., Brown R., Melnyk B.M. (2023). Evidence-based practice improves patient outcomes and healthcare system return on investment: Findings from a scoping review. Worldviews Evid.-Based Nurs..

[2] Melnyk B.M., Gallagher-Ford L., Zellefrow C., Tucker S., Thomas B., Sinnott L.T., Tan A. (2018). The first U.S. study on nurses’ evidence-based practice competencies indicates major deficits that threaten healthcare quality, safety, and patient outcomes. Worldviews Evid.-Based Nurs..

[3] Saifan A., Devadas B., Mekkawi M., Amoor H., Matizha P., James J., Al-Yateem N. (2021). Managing the theory-practice gap in nursing education and practice: Hearing the voices of nursing students in the United Arab Emirates. J. Nurs. Manag..

[4] Shayan S.J., Kiwanuka F., Nakaye Z. (2019). Barriers associated with evidence-based practice among nurses in low- and middle-income countries: A systematic review. Worldviews Evid.-Based Nurs..

[5] Fu L., Chang Y., Wang L., Chao Y. (2020). Barriers and facilitators of implementing evidence-based practice in nursing: A systematic review. Int. J. Nurs. Pract..

[6] Graham I.D., Logan J., Harrison M.B., Straus S.E., Tetroe J., Caswell W., Robinson N. (2006). Lost in knowledge translation: Time for a map?. J. Contin. Educ. Health Prof..

[7] Torres C.P., Mendes F.J.M., Barbieri-Figueiredo M.C. (2023). Use of the Knowledge-to-Action framework for the implementation of evidence-based nursing in child and family care: Study protocol. PLoS ONE.

[8] Field B., Booth A., Ilott I., Gerrish K. (2019). Using the Knowledge to Action framework in practice: A citation analysis and systematic review. J. Adv. Nurs..

[9] Hailemariam M., Bustos T., Montgomery B., Barajas R., Evans L.B., Drahota A. (2019). Evidence-based intervention sustainability strategies: A systematic review. Implement. Sci..

[10] Lennox L., Maher L., Reed J. (2018). Navigating the sustainability landscape: A systematic review of sustainability approaches in healthcare. Implement. Sci..

[11] Shelton R.C., Cooper B.R., Stirman S.W. (2018). The Sustainability of Evidence-Based Interventions and Practices in Public Health and Health Care. Annu. Rev. Public Health.

[12] Coyne I., Holmström I., Söderbäck M. (2018). Centeredness in healthcare: A concept synthesis of family-centered care, person-centered care and child-centered care. J. Pediatr. Nurs..

[13] Torres C., Mendes F., Duarte A.M., Vilaça S., Barbieri-Figueiredo M.C. (2024). Implementation of evidence-based practice in paediatric nursing care: Facilitators and barriers. Collegian.

[14] Tong A., Sainsbury P., Craig J. (2007). Consolidated criteria for reporting qualitative research (COREQ): A 32-item checklist for interviews and focus groups. Int. J. Qual. Health Care.

[15] Nilsen P., Bernhardsson S. (2019). Context matters in implementation science: A scoping review of determinant frameworks that describe contextual determinants for implementation outcomes. BMC Health Serv. Res..

[16] Wei H., Sewell K.A., Woody G., Rose M.A. (2018). The state of the science of nurse work environments in the United States: A systematic review. Int. J. Nurs. Sci..

[17] Melnyk B.M., Gallagher-Ford L., Long L.E., Fineout-Overholt E. (2014). The establishment of evidence-based practice competencies for practicing registered nurses and advanced practice nurses in real-world clinical settings: Proficiencies to improve healthcare quality, reliability, patient outcomes, and costs. Worldviews Evid.-Based Nurs..

[18] Bardin L. (2016). Análise de Conteúdo.

[19] Skivington K., Matthews L., Simpson S.A., Craig P., Baird J., Blazeby J.M., Boyd K.A., Craig N., French D.P., McIntosh E. (2021). A new framework for developing and evaluating complex interventions: Update of Medical Research Council guidance. BMJ.

[20] Melnyk B.M., Fineout-Overholt E. (2023). Evidence-Based Practice in Nursing & Healthcare: A Guide to Best Practice.

[21] Graves B.A., O’Neal P.V., Roussel L., Polancich S. (2018). EBP design and translation: Teaching how to begin a scholarly practice project. Worldviews Evid. Based Nurs..

[22] Saunders H., Stevens K.R., Vehviläinen-Julkunen K. (2016). Nurses’ readiness for evidence-based practice at Finnish university hospitals: A national survey. J. Adv. Nurs..

[23] Aarons G.A., Ehrhart M.G., Farahnak L.R., Sklar M. (2017). Aligning leadership across systems and organizations to develop a strategic climate for evidence-based practice implementation. Annu. Rev. Public Health.

[24] Mathisen C., Heyn L.G., Jacobsen T.-I., Bjørk I.T., Hansen E.H. (2022). The use of practice education facilitators to strengthen the clinical learning environment for nursing students: A realist review. Int. J. Nurs. Stud..

